# Control efficacy of *Bidens pilosa* L. extract against immature stages of *Musca domestica* L. (Diptera: Muscidae): laboratory and semi-field evaluations

**DOI:** 10.7717/peerj.21544

**Published:** 2026-07-17

**Authors:** Waranya Ardburai, Duangrat Thongphak, Ubon Tangkawanit

**Affiliations:** Department of Entomology and Plant Pathology, Khon Kaen University, Khon Kaen, Thailand

**Keywords:** Plant extract, House fly, GSTs, CarE, Bioactive compound

## Abstract

The development of environmentally friendly insecticides is crucial for managing house fly (*Musca domestica*) populations in urban and agricultural settings. This study evaluated the insecticidal potential of *Bidens pilosa* extracts against the immature stages of house flies. In the laboratory, extracts using solvents of varying polarity were tested against eggs, larvae, and pupae. The insecticidal activity of *B. pilosa* extracts varied by solvent and life stage. Hexane and ethyl acetate extracts were the most effective ovicides and showed high larvicidal toxicity upon ingestion. In contrast, acetone and ethanol extracts acted as potent contact larvicides, while the hexane extract was also significantly pupicidal. Notably, extracts with lower lethal effects still demonstrated significant sub-lethal impacts by affecting the insect growth. The ethyl acetate extract, identified as the most effective, was evaluated for its impact on detoxifying enzyme activities. This extract was found to significantly inhibit glutathione-S-transferase (GSTs) activity in treated larvae but did not affect carboxylesterase (CarE). A subsequent field trial on a poultry farm using the ethyl acetate extract resulted in a 75.71% reduction in the house fly population within three days. Gas chromatography-mass spectrometry (GC-MS) analysis identified linolenic acid as a major compound. These findings demonstrate that *B. pilosa* extract, particularly the ethyl acetate fraction, is a promising botanical insecticide for effective house fly control.

## Introduction

The house fly (*Musca domestica* Linnaeus, 1758) is a cosmopolitan, significant medical and veterinary pest causing irritation to humans and livestock (De Jonge et al., 2020). Although it does not bite, it serves as a mechanical vector for over 100 species of pathogens (Morey & Khandagle, 2012), causing annual economic losses reaching 1 billion USD in the United States (Geden et al., 2021). House flies exhibit a high reproductive rate, with a single female depositing up to 900 eggs in her lifespan (West, 1951; Geden et al., 2021). Controlling them at the localized, immature stage in unsanitary areas is crucial for effective management (Hinkle & Hogsette, 2021).

Synthetic insecticides remain the principal control strategy due to their rapid action and local availability (Aktar, Sengupta & Chowdhury, 2009). However, chemical residues cause environmental contamination, disrupt ecosystem balance, and harm non-target organisms, which can trigger pest resurgence (Denholm & Devine, 2013; Sánchez-Bayo, 2021; Barathi et al., 2024). Furthermore, chemical exposure poses short-and long-term health risks to humans and animals (Ahmad et al., 2024). Crucially, continuous application over the past eight decades has driven widespread insecticide resistance in insect populations (Barathi et al., 2024; Pu & Chung, 2024). Consequently, developing alternative eco-friendly strategies like biopesticides is necessary.

Plant-derived biopesticides are environmentally safe, biodegradable, and less hazardous than conventional chemicals (Godlewska, Stepnowski & Pałzkiewicz, 2020). Unlike single-compound synthetic insecticides that exert high selection pressure, the multi-component nature of plant extracts provides multiple modes of action, delaying resistance development (Rattan, 2010). These plant extracts can act as growth inhibitors, repellents, antifeedants, or oviposition deterrents against dipterans (Koul, 2005). Various plant extracts, such as *Abrus precatorius* L. and *Solanum trilobatum* L., has been reported for controlling dipteran population (Muthukrishnan, Pushpalatha & Kasthuribhai, 1997). Plants in the Asteraceae family possess high insecticidal activity due to diverse phytochemicals, including phenolic acids, terpenoids, and polysaccharides (Koc et al., 2015; Czerniewicz, Sytykiewicz & Chrzanowski, 2024). *Bidens pilosa*, an invasive Asteraceae weed with high reproductive and adaptive capacities, is considered a noxious weed in over 40 countries, reducing crop yields *via* competition and allelopathy (Holm et al., 1977; Silva et al., 2011; El-Gawad, 2016; Nguyen et al., 2019; Rokaya et al., 2012; Kato-Noguchi & Kurniadie, 2024).

*Bidens pilosa* L. exhibits insecticidal activity against various agricultural, stored-product, and public health pests (Ahmed et al., 2021; Li et al., 2021a; Li et al., 2021b). This bioactivity is linked to diverse compounds, including flavonoids, fatty acids, terpenes, and linolenic acid (Geissberger & Séquin, 1991; Xuan & Khanh, 2016; Singh et al., 2017; Son, Tuan & Tran, 2022). Since phytochemical yields depend heavily on the extraction solvent (Abubakar & Haque, 2020), utilizing this unwanted agricultural weed as a biopesticide source represents a highly beneficial strategy.

Detoxification enzymes play a critical role in metabolizing both synthetic pesticides and natural plant secondary metabolites (Ramsey et al., 2010). This insect system is divided into three phases: Phase I modification such as oxidation, hydrolysis and reduction (primarily cytochrome P450s and carboxylesterases, CarE), Phase II conjugation (glutathione S-transferases, GSTs), and Phase III transport (ATP-binding cassette transporters) (Li, Yu & Feng, 2019). Baseline enzyme activity directly dictates insecticide susceptibility and resistance evolution (Fan et al., 2023). Although *B. pilosa* extracts can modulate detoxification enzymes such as inhibiting GSTs in *Plutella xylostella* (Li et al., 2021a) or affecting GSTs and CarE in *Apis mellifera* (Tang et al., 2023), its insecticidal properties against immature *M. domestica* and its subsequent effects on this species’ detoxification enzymes have not yet been reported.

The main objectives of this research were to evaluate the insecticidal activity of *B. pilosa* extracts, prepared using various solvents, against the immature stages of *M. domestica* under laboratory and farm conditions; to identify the chemical constituents of the most effective extract using gas chromatography-mass spectrometry (GC-MS); and to investigate the extract’s sublethal effects on key Phase I (CarE) and Phase II (GSTs) detoxification enzymes.

## Materials & Methods

### *Musca domestica* rearing

A house fly (*Musca domestica*) colony was maintained at the Department of Entomology, Khon Kaen University, under controlled laboratory conditions of 28 ± 2 °C, 60 ± 10% relative humidity (RH), and a 12:12 h light-dark photoperiod.

Adult flies were housed in mesh cages and provided a diet consisting of a 1:3 (w/w) mixture of sucrose and powdered milk (Tangkawanit, Kaewkamsan & Siri, 2018). For oviposition, a petri dish containing fish meal on moist tissue paper was supplied as a substrate.

Following oviposition, egg masses were transferred into a larval rearing container (18  × 27  × 12 cm) filled with a medium composed of water, rice bran, and chicken feed (Ardburi & Tangkawanit, 2022). Once the larvae developed to the third instar (characterized by a noticeably stouter body and a length of 8–12 mm), they were separated from the rearing medium using a sieve. After passing through the sieve, mature larvae descended into a dry container below to begin their pupal development. The pupae were moved to a new cage in preparation for adult emergence. House flies from at least the second generation were used in the experiment.

### Preparation of *Bidens pilosa* extracts

Arial parts of *B. pilosa* were extracted using the maceration method due to its simplicity and ability to preserve heat-sensitive bioactive compounds by avoiding high temperatures, unlike Soxhlet extraction. Cold pressing was not employed as it is primarily effective for oil extraction rather than broad phytochemical profiles (Azwanida, 2015). Since the target compounds for house fly control are unknown, sequential extraction was performed using solvents of increasing polarity (Non-polar to highly polar) hexane (98%, Zenith Science Co., Ltd.), ethyl acetate (98%, Zenith Science Co., Ltd.), acetone (99.8%, Zenith Science Co., Ltd.), ethanol (95%, Zenith Science Co., Ltd.), and distilled water. This approach allows efficient separation of a broad range of phytochemicals based on polarity, maximizing the recovery of diverse bioactive constituents and providing a comprehensive chemical profile of the plant material.

A five-kilogram arial part of *B. pilosa* was cleaned, chopped into small pieces, and sun-dried for 3–5 days at 33 °C ± 2 °C, then, they were ground into fine powder. Plant powder was macerated in hexane (1:3) (plant: hexane). After 72 h, the supernatant was collected by filtering through filter paper (Whatman No. 1). The solvent in the supernatant evaporated in a rotary evaporator. The remaining material after rotary evaporation was transferred into an amber glass bottle and stored in the refrigerator at 4 °C until use. The remaining plant residue from hexane extraction was then extracted with ethyl acetate, acetone, ethanol, and distilled water, respectively. Stock solutions (100 mg/mL) were prepared for each solvent extraction. For non-polar solvent extraction, one g of crude plant extract was first dissolved in 0.4 mL of acetone (99%, Fisher scientific Co., Ltd.) and then added to 0.05% or 0.005 mL emulsifier (Tween 80, Ruam Chemistry1986 Co., Ltd), then added to 9.595 mL of RO water for stock solution (the concentration was modified from Muhammed et al., 2022).

### Bioassay

A Completely Randomized Design (CRD) was used due to the homogeneity of experimental units and simplicity of implementation. The treatments included *B. pilosa* extracts prepared in five different solvents (hexane, ethyl acetate, acetone, ethanol, and distilled water), along with two control treatments: 4% acetone and distilled water. The experiment was conducted with five replications. The maximum test concentration (100 mg/mL) was selected as an initial screening dose to evaluate the full insecticidal potential of the plant extract, since plant-derived extracts may vary considerably in toxicity and some may exhibit relatively low bioactivity. This preliminary high-dose assessment was used to determine whether the extract possessed sufficient biological efficacy to justify further dose–response evaluation. After significant mortality was observed at 100 mg/mL, the extract was considered sufficiently promising for subsequent bioassays using lower concentrations to establish the effective bioactive range and to calculate lethal concentration values (LC_50_ and LC_90_). LC_50_ and LC_90_ were evaluated at the concentration of 0, 25, 50, 75, and 100% using Probit analysis (Finney, 1971). The concentration range was selected based on a preliminary screening where 100 mg/mL demonstrated maximum efficacy, allowing for a systematic evaluation of descending concentrations.

The mortality data for each treatment was adjusted (Abbott, 1925) using Abbott’s formula.

\begin{eqnarray*}Corrected~mortality= \frac{\%~mortality~in~treatment-\%~mortality~in~control}{100-\%~mortality~in~control} \times 100 \end{eqnarray*}

### Egg (contact toxicity)

Twenty newly oviposited eggs of house fly (<6 h old) were transferred into a larval food container (2 × 5 cm). 200 µl of 100 mg/mL *B. pilosa* extract was dropped onto the eggs using a micropipette. The newly hatched larvae from this experiment were transferred to a new larval food container to observe their development. The egg hatching percentage (Nisar et al., 2021) was calculated.

### Larva

**Contact toxicity:** Testing for contact toxicity was performed using the dipping method, which was modified from Abdel, Abdel & Razik (2017). Ten larvae (2 days old) were placed into a cotton muslin bag (3  × 4 cm) tied with a rope, and attached to a string; the bag was then dipped into a container containing 5 mL of *B. pilosa* extracts (100 mg/mL) for 40 s. The larvae were then transferred to a larval food container (2 cm in height × 5 cm in diameter), and their development was observed daily until adult emergence.

**Feeding activity:** The experiment was modified from Subaharan et al. (2021). Five grams of larval food were placed in a larval food container (2 cm high by 5 cm in diameter) that was filled with water, rice bran, and chicken feed (Ardburi & Tangkawanit, 2022). 2.5 mL of *B. pilosa* extract (100 mg/mL) or control were sprayed onto each larval food. Ten house fly larvae (2 days old) were transferred into the larval food. The number of larval deaths was recorded every 24 h for 3 days. The remaining larvae in the experiment were transferred to a new food container to monitor their development.

### Pupa (contact toxicity)

Toxicity was investigated using the dipping method. A cotton muslin bag (3 × 4 cm) tied with a rope, and attached to a string and containing 10 house fly pupae (3 days old) was dipped into 5 mL of *B. pilosa* extract (100 mg/mL) or control for 40 s. The pupae were then moved to a plastic container (7 × 9  ×  4 cm). The adult emergence rate and adult sex ratio were recorded every 24 h for 5 days.

### Data analysis

All data were analyzed using a one-way Analysis of Variance (ANOVA) to determine the overall effect of the treatments. Prior to analysis, the data were examined for normality and homogeneity of variance. When the overall ANOVA F-test indicated significant differences (*p* < 0.05), Tukey’s Honestly Significant Difference (HSD) test was performed for *post-hoc* multiple comparisons.

All statistical analyses were conducted using the Statistix 10 program (Analytical Software, 2024). Probit analysis (Finney, 1971) in SPSS was used to estimate LC_5__0_ and LC_9__0_ values from the 72 h mortality data.

### Analysis of bioactive compound of *B. pilosa* extract

The chemical composition of the three potentially best extracts of *B. pilosa* in three solvents (hexane, ethyl acetate, and ethanol) was analyzed by gas chromatography-mass spectrometry analysis. which demonstrated the highest overall potency against house fly immature stages, was prioritized for detailed compound identification.

The analysis employed an Agilent 7890A, column HP-5 capillary column (20 m × 0.18 mm, 0.18 um), MS Agilent 7000. Helium at a rate of one mL/min was used as carrier gas and 2 µL sample volume was injected. The program started at 40 °C, held for 5 min, ramped at 8 °C/min to 200 °C, then ramped at 5 °C/min to 280 °C, and held for 20 min adapted from Olivia, Googness & Obinna (2021), then save the result as a chromatogram. Compounds were identified by comparing their mass spectra and retention indices with data stored in the NIST MS Search 2.0 spectral library.

The results were evaluated in the library of NIST MS Search 2.0. This experiment was analyzed at the Center for Scientific and Technological Equipment, Suranaree University of Technology.

### Enzyme activity

The detoxifying enzyme activity of glutathione-S-transferase (GSTs) and carboxylesterase (CarE) were assessed in ethyl acetate extracts of *B. pilosa*. Newly hatched house fly larvae (<24 h) was reared on larval food mixed with *B. pilosa* extract (at LC_90_ concentration) until the 3rd larval stage. Twenty five larvae of the third larval stage were prepared for enzyme testing activity test. The head was cut off from the body, and the body portion was homogenized in 0.5 mL of buffer (pH 7.2, 0.1 M phosphate buffer, and 1% Triton X–100). The homogenate was centrifuged for 10 min at 4 °C at 6,000 rpm (3,823.6×g), and the supernatants were utilized as sources of enzymes (Kumrungsee et al., 2014).

GSTs were measured by adding 200 µl of the enzyme source to 1,150 µl of 0.1 M potassium phosphate buffer (pH 7.3), then 10 µl of 0.15 M 1-choro-2,4-dinitrobenzene was added. After preparation, spectrophotometer was used to measure the reaction progress at intervals of 30 s at 25 °C and 340 nm wavelength.

CarE was determined by adding 50 µl of 0.12 M p-nitro phenylacetate to the enzyme source (200 µl), followed by 2.9 mL of 0.1 M phosphate buffer (pH 7.5). A micro-plate reader was used to measure the reaction progress for 3 min at 25 °C at 400 nm (Kumrungsee et al., 2014).

The experiment for GSTs and CarE enzyme activity was performed with five replications.

### Toxicity test to house flies in the poultry farm

**Figure 1:**
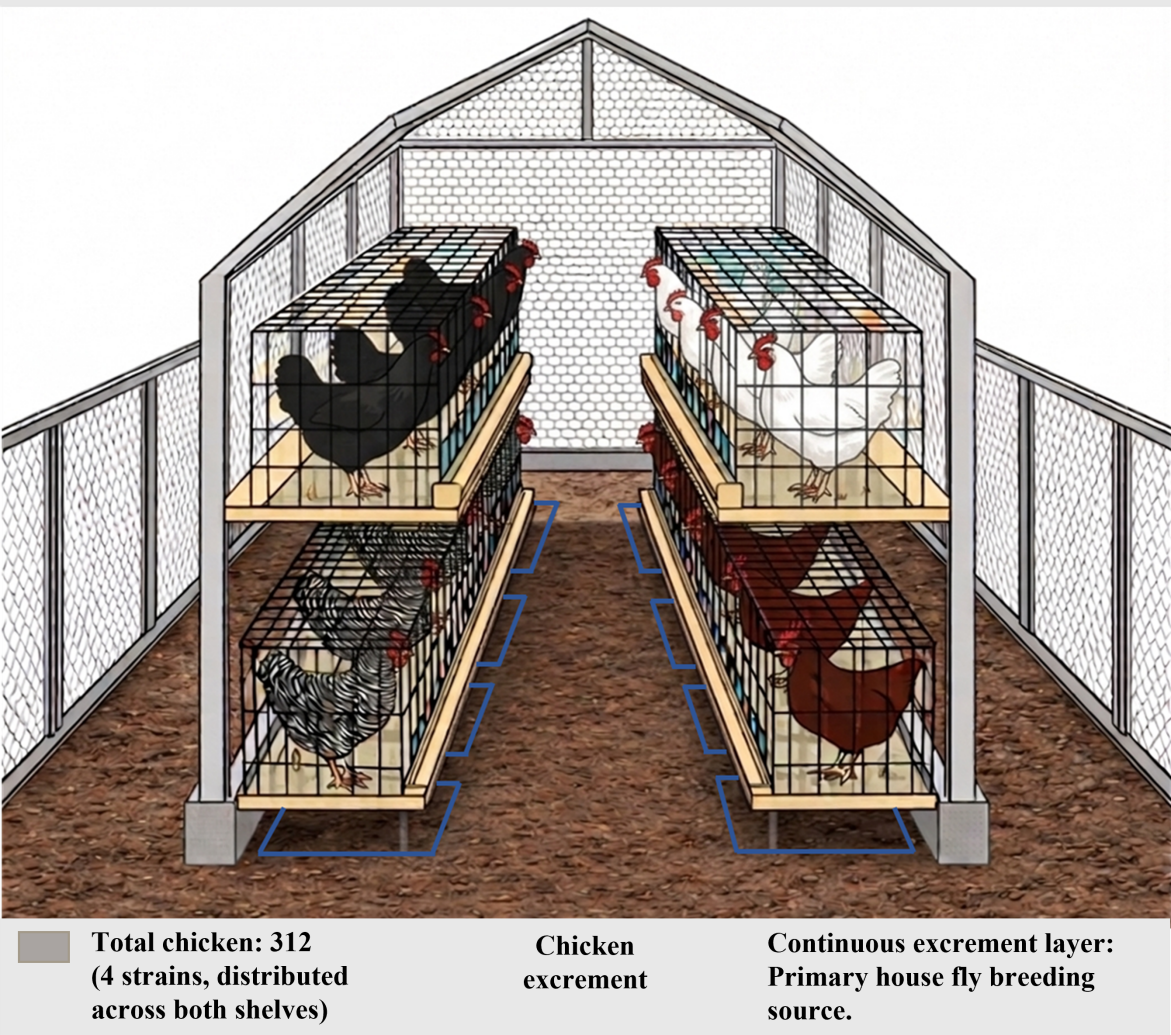
Toxicity test to house flies in the poultry farm. The experiment involved a cage housing chickens, with chicken manure on the floor below serving as the breeding ground for the house fly colony. Quadrats of 50 × 50 cm were designated as experimental units, with a 100 cm gap between plots.

The LC_90_ value from the feeding activity experiment was assessed for house fly control on the Department of Animal Science’s poultry farm, Faculty of Agriculture, Khon Kaen University. The experiment was carried out in an open rearing house with dimensions of 6 × 50  ×  2 m. The lower portion of the house was built with concrete, while the upper part was enclosed with a wire mesh extending to the ceiling. There were two long shelves in the poultry house with a total of 312 chickens (four strains). Each chicken was raised in a small cage on the shelf in the rearing house. On the left side, Super Haoka chickens were in the upper cages, and Barred Plymouth Rock chickens were in the lower cages. On the right side, White Leghorn stayed in the cage above, and Rhode Island Red chickens were in the lower cages. Food and water were provided in the cage every day. On the floor beneath, chicken excrement was the breeding source for the house fly breeding colony (Fig. 1). During the experiment, the temperature was 29–32 °C and the humidity was 65–76%.

A paired-sample *t*-test was applied to the data from the four trials conducted on the floor beneath the chicken cage, each consisting of a treatment plot and a control plot. To survey the insects, 50 × 50 cm quadrats were used. There was 100 cm between each plot.

In each treatment plot, 250 mL of *B. pilosa* extract was sprayed at a concentration of LC_90_, whereas in the control plots, 250 mL of distilled water was sprayed using a handheld pump-pressure sprayer (droplet size approximately 400 µm). The house fly’s immature stage density was determined at the start of the experiment as well as one, three, five, and seven days after application. Percentage reduction was calculated by using Henderson and Tilton’s formula as follows.

\begin{eqnarray*}\%~reduction=(1- \frac{n~in~Control~before~treatment\times n~in~Treatment~after~treatment}{n~in~Control~after~treatment\times n~in~Treatment~before~treatment} )\times 100 \end{eqnarray*}

This work was performed according to the Guidelines for Animal Experimentation of the National Research Council of Thailand. It approved a number of IACUC-KKU-51/68 by the Animal Ethics Committee of Khon Kaen University, Thailand. All efforts were made to minimize animal suffering. The poultry farm field trial was conducted in accordance with institutional guidelines for the ethical use of animals in research. No invasive or harmful procedures were employed, and all measures were taken to minimize any potential disturbance to the animals throughout the experimental period.

## Results

### Influence of extraction solvents on toxicity

The toxicological efficacy and sublethal developmental impacts of *B. pilosa* extracts against the immature stages of house fly, evaluated across various solvent systems under laboratory was systematically compiled and presented in Tables 1, 2, 3 and 4.

**Table 1:** Mortality rate and development of house fly eggs. Ovicidal activity and subsequent developmental effects of different solvent extracts from *Bidens pilosa* (100 mg/mL) against the house fly, *Musca domestica*.

**Treatments** ^**1/**^	**Accumulated eggs hatching** ^**2/,3/**^		**% eggs hatching**	**% Total mortality**	**Corrected mortality**	**Number of pupae**	**No. adult**	**% Total corrected mortality**	**Sex ratio** **M:F**	**Larval** **Duration** time (day)	**Pupal** **Duration time (day)**
	**24 hr**	**48 hr**									
Hexane	0 ± 0^d^	0 ± 0^d^	0	100	100	–	–	100	–	–	–
Ethyl acetate	0 ± 0^d^	0 ± 0^d^	0	100	100	–	–	100	–	–	–
Acetone	4.6 ± 2.19^c^	4.6 ± 2.19^c^	23	77	73.56	2.2 ± 1.64^c^	2.2 ± 1.64^c^	85.52	1:0.83	5.50 ± 0.46^a^	5 ± 0^c^
Ethanol	3 ± 2.73^c^	3 ± 2.73^c^	15	85	82.76	0.6 ± 0.54^d^	0.6 ± 0.54^d^	96.05	3:0	3.20 ± 2.94^b^	5 ± 0^c^
Distilled water	11.6 ± 0.54^b^	11.6 ± 0.54^b^	58	42	33.33	9.4 ± 0.54^b^	7.4 ± 0.54^b^	51.31	1:0.37	5.38 ± 0.13^a^	5.63 ± 0.07^a^
Control (4% acetone)	16 ± 1.22^a^	17.2 ± 0.44^a^	86	14	1.15	16.4 ± 1.14^a^	14.2 ± 0.44^a^	6.58	1:1.62	5.36 ± 0.07^a^	5.28 ± 0.15^b^
Control (water)	17.2 ± 0.44^a^	17.4 ± 0.54^a^	87	13	0	17 ± 0.70^a^	15.2 ± 1.09^a^	0	1:1.53	5.32 ± 0.06^a^	5.32 ± 0.15^b^
F-value	132.29	155.78	–	–	–	405.44	333.39	–	–	25.40	4,427.60
df (treatment, errror)	6, 28	6, 28	–	–	–	6, 28	6, 28	–	–	6, 28	6, 28
*P*-value	0.0000	0.0000	–	–	–	0.0000	0.0000	–	–	0.0000	0.0000
CV	19.09	17.80	–	–	–	13.10	14.48	–	–	31.94	2.30

**Notes.**

^1/^ *n* = 20, ^2/^ Mean ± SD, ^3/^ Within each column, Mean ± SD followed by the same small letter indicate not significantly different according to Fisher’s Least Significant Difference (LSD) (*P* ≥ 0.05).

**Table 2:** Mortality rate and development of house fly larvae. Larvicidal activity and developmental effects of *Bidens pilosa* solvent extracts (100 mg/mL) against *Musca domestica* larvae using the feeding and dipping method.

**Treatments** ^**1/**^	**Cumulative mortality rate** ^**2/,3/**^			**% Total mortality**	**Corrected mortality**	**Number of pupae**	**No.** **adult**	**% Total corrected mortality**	
	**24 hr**	**48 hr**	**72 hr**						
Dipping method									
Hexane	2 ± 1.22^a^	2.4 ± 0.89^b^	2.4 ± 0.89^c^	24	22.44	7.4 ± 0.54^b^	6.4 ± 0.89^b^	30.43	
Ethyl acetate	2.8 ± 0.44^a^	2.8 ± 0.44^b^	3.8 ± 0.44^b^	38	36.73	6.2 ± 0.44^c^	5.6 ± 0.89^b^	39.13	
Acetone	3 ± 1.4^a^	4 ± 1.4^a^	7 ± 1.4^a^	70	69.38	3 ± 1.4^d^	2.8 ± 1.30^c^	69.56	
Ethanol	3 ± 1.4^a^	4.6 ± 0.89^a^	7.2 ± 1.30^a^	72	71.42	2.8 ± 1.30^d^	1.8 ± 1.09^c^	80.43	
Distilled water	0 ± 0^b^	0 ± 0^c^	1.2 ± 1.09^cd^	12	10.20	8.8 ± 1.09^a^	6.6 ± 1.34^b^	28.26	
Control (4% acetone)	0 ± 0^b^	0.2 ± 0.44^c^	0.2 ± 0.44^d^	2	0	9.4 ± 0.54^a^	9 ± 1.22^a^	2.17	
Control (water)	0 ± 0^b^	0.2 ± 0.44^c^	0.2 ± 0.44^d^	2	0	9.6 ± 0.54^a^	9.2 ± 0.83^a^	0	
F-value	13.49	30.60	49.46	–	–	48.36	32.77	-	
df	6	6, 28	6, 28	–	–	6, 28	6, 28	-	
*P*-value	0.0000	0.0000	0.0000	–	–	0.0000	0.0000	–	
CV	58.49	38.18	30.19	–	–	13.73	18.63	–	
Feeding method									
Hexane	10 ± 0^a^	10 ± 0^a^	10 ± 0^a^	100	100	–	–	100	
Ethyl acetate	8.2 ± 1.30^b^	10 ± 0^a^	10 ± 0^a^	100	100	–	–	100	
Acetone	7.6 ± 0.54^b^	7.8 ± 0.44^b^	8.4 ± 0.89^ab^	84	83.33	1.2 ± 0.44^b^	0.4 ± 0.54^cd^	95.83	
Ethanol	8.4 ± 2.30^b^	8.6 ± 2.19^b^	8.8 ± 1.78^ab^	88	87.5	1.2 ± 1.78^b^	0.8 ± 1.09^bc^	91.66	
Distilled water	0.6 ± 0.54^c^	0.6 ± 0.54^c^	7.2 ± 3.03^b^	72	70.83	1.6 ± 0.89^b^	1.2 ± 0.44^b^	87.50	
Control (4%acetone)	0 ± 0^c^	0 ± 0^c^	0.4 ± 0.54^c^	4	0	9.6 ± 0.54^a^	9 ± 0.70^a^	6.25	
Control (water)	0 ± 0^c^	0 ± 0^c^	0.4 ± 0.54^c^	4	0	9.6 ± 0.54^a^	9.6 ± 0.54^a^	0	
F-value	94.34	153.64	45.78	–	–	137.17	262.27	–	
df (treatment, errror)	6, 28	6, 28	6, 28	–	–	6, 28	6, 28	–	
*P*-value	0.0000	0.0000	0.0000	–	–	0.0000	0.0000	–	
CV	20.96	16.46	21.74	–	–	24.99	19.92	–	

**Notes.**

^1/^ *n* = 10, ^2/^ Mean ± SD, ^3/^ Within each column in the same method, Mean ± SD followed by the same small letter indicate not significantly different according to Fisher’s Least Significant Difference (LSD) (*P* ≥ 0.05).

**Table 3:** Mortality rate of house fly pupae tested with *Bidens pilosa* extracts (100 mg/mL).

**Treatments** ^**1/**^	**Accumulated** adult emergence^**2/,3/**^					**No. adult**	**Age of pupa (days)**	**% Total mortality**	**Corrected mortality**	**Sex ratio** **M:F**	**Pupal** **Duration time** (day)
	**24 hr**	**48 hr**	**72 hr**	**96 hr**	**120 hr**						
Hexane	0 ± 0	0 ± 0	0 ± 0	0 ± 0^e^	0 ± 0^d^	0 ± 0^d^	–	100	100	–	–
Ethyl acetate	0 ± 0	0 ± 0	0 ± 0	0 ± 0^e^	0.6 ± 0.54^d^	0.6 ± 0.54^d^	8	94	93.18	1:2	8 ± 0^a^
Acetone	0 ± 0	0 ± 0	0 ± 0	0.6 ± 0.54^de^	0.6 ± 0.54^d^	0.6 ± 0.54^d^	7-8	94	93.18	3:0	7.6 ± 0.54^b^
Ethanol	0 ± 0	0 ± 0	0 ± 0	1 ± 0^d^	1.6 ± 0.54^c^	1.6 ± 0.54^c^	7-8	84	81.81	1:0.6	7.5 ± 0.70^b^
Distilled water	0 ± 0	0 ± 0	0 ± 0	2.6 ± 0.54^c^	2.8 ± 0.44^b^	2.8 ± 0.44^b^	7-8	72	68.18	1:0.75	7.66 ± 0.23^ab^
Control (4% acetone)	0 ± 0	0 ± 0	0 ± 0	4.6 ± 0.54^b^	8.6 ± 0.54^a^	8.6 ± 0.54^a^	7-8	14	2.27	1:1.86	7.65 ± 0.02^ab^
Control (water)	0 ± 0	0 ± 0	0 ± 0	5.8 ± 0.83^a^	8.8 ± 0.44^a^	8.8 ± 0.44^a^	7-8	12	0	1:1.93	7.65 ± 0.01^ab^
F-value	–	–	–	118.38	317.00	317.00	–	–	–	–	487.58
df (treatment, errror)	–	–	–	6, 28	6, 28	6, 28	–	–	–	–	6, 28
*P*-value	–	–	–	0.0000	0.0000	0.0000	–	–	–	–	0.0000
CV	–	–	–	22.92	14.55	14.55	–	–	–	–	4.47

**Notes.**

^1/^n=10, ^2/^Mean ± SD, ^3/^ Within each column, Mean ± SD followed by the same small letter indicate not significantly different according to Fisher’s Least Significant Difference (LSD) (*P* ≥ 0.05).

**Table 4:** Lethal concentration (LC_50_, LC_90_) of *Bidens pilosa* extract against house flies.

**Stage**	**Method**	**Solvent**	***n***	**Slope ± SE**	**LC** ${}_{\mathbf{50}}^{\mathbf{1/}}$ ** (mg/mL)**	**LC**_**90**_ (**mg/mL)**	**95% confidence limits**	**Chi-square**
Eggs	Spaying	Hexane	100	2.06 ± 0.30	27.50	115.11	1.46–2.66	0.78
		Ethyl acetate	100	2.79 ± 1.08	6.80	19.56	0.67–4.92	1.03
Larvae	Dipping	Acetone	50	1.40 ± 0.29	21.37	175.49	0.82–1.98	1.19
		Ethanol	50	1.15 ± 0.29	19.96	255.97	0.58–1.72	1.13
	Feeding	Hexane	50	7.06 ± 1.33	23.32	35.42	4.44–9.68	1.00
		Ethly acetate	50	4.02 ± 1.57	11.10	23.13	0.93–7.10	0.48
Pupa	Dipping	Hexane	50	4.17 ± 0.46	25.48	51.66	3.25–5.09	8.55

**Notes.**

^1/^ Lethal concentrations were determined using probit analysis.

### Ovicidal activity

The findings revealed that the effectiveness of *B. pilosa* extracts in inducing house fly egg mortality varied based on the solvent used (Table 1). Extracts prepared with hexane and ethyl acetate demonstrated the highest efficacy, achieving 100% mortality, while ethanol-based extracts showed slightly lower effectiveness.

In all treatments, surviving house flies were monitored during their growth (Table 1). *Bidens pilosa* extracts in acetone, ethanol, and distilled water had low ovicidal activity, but they had a significantly lower number of surviving pupae when compared to the control (*F* = 405.44, *df* = 6, 28, *P* < 0.01) (Table 1). The results showed that *B. pilosa* extracts from all solvents had no effect on larval developmental time, except for the ethanol extract, which shortened the larval period (*F* = 25.4, *df* = 6, 28, *P* < 0.01) (Table 1). However, extracts from all solvents influenced pupal development, resulting in slightly shorter durations compared to the control (*F* = 4, 427.61, *df* = 6, 28, *P* < 0.01). Additionally, a higher proportion of males than females was found in the trials.

Lethal concentrations (LC_50_ and LC_90_) were evaluated for the *B. pilosa* extracts in hexane and ethyl acetate solvents, which had the most effective ovicidal activity. LC_50_ were 27.50 and 6.80 mg/mL, for hexane and ethyl acetate extracts respectively. LC_90_ were 115.11 and 19.96 mg/mL, respectively (Table 4).

### Larvicidal activity (dipping method)

The results indicated that the ethanol and acetone extracts of *B. pilosa* induced the highest significant larval mortality after 72 h, reaching 72% and 70%, respectively (*F* = 49.46; *df* = 6, 28; *P* < 0.001) (Table 2). In contrast, the distilled water and hexane extracts produced much lower larval mortality rates of 12% and 24%, respectively, while the negative controls (4% acetone and water) showed negligible mortality (2%).

The sublethal effects of the extracts on the surviving larvae significantly impacted their subsequent development. Treatments with ethanol and acetone extracts drastically suppressed the number of surviving individuals that successfully transitioned to the next stages, resulting in the lowest number of pupae (2.8 ± 1.30 and 3 ± 1.40, respectively; *F* = 48.36; *df* = 6, 28; *P* < 0.001) and the lowest adult emergence (1.8 ± 1.09 and 2.8 ± 1.30, respectively; *F* = 32.77; *df* = 6, 28; *P* < 0.001), compared to the control groups.

For the high-efficiency treatment of house fly larvae with *B. pilosa* extracts (Acetone and Ethanol), lethal doses (LC_50_ and LC_90_) were estimated. The results showed that the LC_50_ of those two treatments was 21.37 and 19.96 mg/mL, respectively. LC_90_ were 175.49 and 255.97 mg/mL, respectively (Table 4).

### Larvicidal activity (feeding method)

The feeding method bioassay revealed that *B. pilosa* extracts in hexane and ethyl acetate induced complete larval mortality (100%) after 72 h (Table 2). Although the acetone and ethanol extracts resulted in slightly lower larval mortality rates of 84% and 88%, respectively, their toxic effects were still highly significant when compared to the negative controls, which exhibited only 4% mortality (*F* = 45.78; *df* = 6, 28; *P* < 0.001).

Regarding developmental effects, the percentage of surviving larvae that successfully emerged into adults was remarkably low in the acetone, ethanol, and distilled water treatment groups.

Lethal concentrations (LC_50_ and LC_90_) were estimated for the high-efficiency treatments in controlling house fly larvae, viz., *B. pilosa* extract in hexane and ethyl acetate solvents. The results showed that the LC_50_ of those two treatments was 23.32 and 11.10, and the LC_90 _ was 35.42 and 23.13 mg/mL, respectively (Table 4).

### Pupicidal activity

*Bidens pilosa* extracts were discovered to have an effect on pupal mortality in several solvents. The results showed that the *B. pilosa* hexane extract was highly efficient causing 100% pupal mortality (Table 3) with an LC_50_ of 25.48 mg/mL and LC_90_ of 51.66 mg/mL (Table 4). The differences between the ethyl acetate and acetone extracts were not statistically significant. Extracts from *B. pilosa* had no effect on the pupal duration, except for the ethyl acetate extract, which extended it by one day compared with the control (*F* = 487.58, *df* = 6, 28, *P* < 0.01). After applying all of the *B. pilosa* extracts, the proportion of adult males was higher than that of females (Table 3).

### Analysis of bioactive compound of *B. pilosa* extract

**Figure 2:**
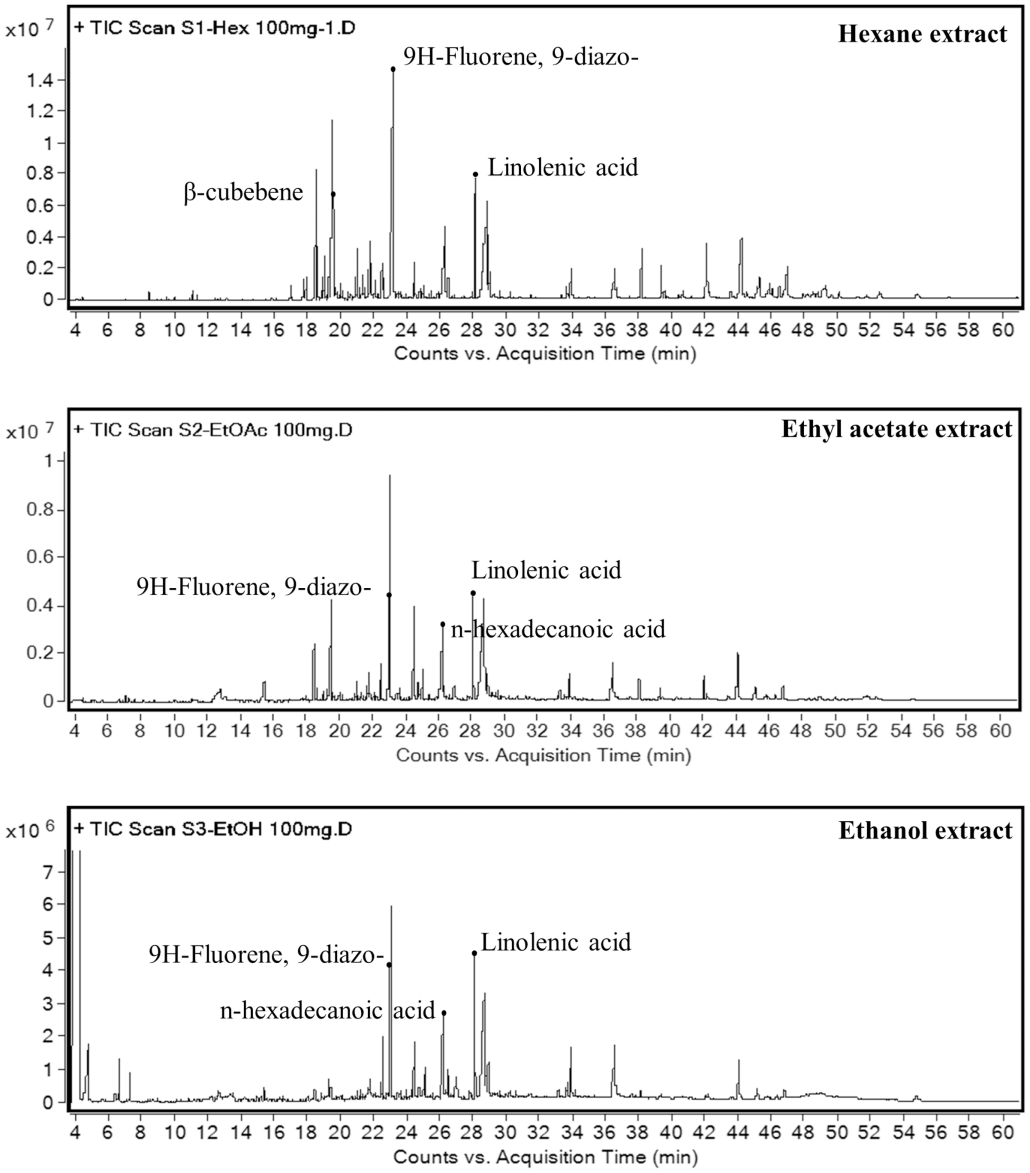
Major compounds of *Bidens pilosa* extracts identified by GC–MS analysis from different solvent extractions: hexane, ethyl acetate, and ethanol extraction.

Hexane, ethyl acetate, and ethanol were selected as the solvents for which *B. pilosa* extracts had the best potential for suppressing houseflies during their immature stages (egg, larval, and pupal stages). GC-MS was utilized to examine the chemical components of these extracts. For the hexane extract, 9H-Fluorene, 9-diazo- (alkene) were the compound present in highest concentration (21.91%), followed by linolenic acid (a fatty acid) at 11.43%, β-cubebene (sesquiterpene) at 9.63%, n-hexadecanoic acid (fatty acid) at 7.33%, β-caryophyllene (sesquiterpene) at 5.43%, and stigmasterol (triterpenes) at 5.35%. Ethyl acetate extracts were composed of three major components: linolenic acid (21.30%), 9H-fluorene, 9-diazo- (alkene) (19.72%), and n-hexadecanoic acid (8.98%). Among the compounds in the ethanol extract, linolenic acid (fatty acid) was present at the highest level 22.72%, followed by 9H-fluorene, 9-diazo-(alkene) (11.83%), n-hexadecanoic acid (11.16%), and phytol (8.31%), respectively (Table 5) (Fig. 2).

**Table 5:** Main bioactive compounds from extract of *Bidens pilosa*.

**Solvents**	**Main compound**	**Class of compound**	**Peak area %**
Hexane	Phytol	Terpene	4.34
	Stigmasterol	Tetracyclic triterpenes	5.35
	β-caryophyllene	Bicyclic sesquiterpene	5.43
	n-hexadecanoic acid	Fatty acid	7.33
	β-cubebene	Sesquiterpenes	9.63
	Linolenic acid	Fatty acids	11.43
	9H-fluorene, 9-diazo-	Alkene	21.91
Ethyl acetate	Phytol	Diterpene	4.44
	Stigmasterol	Tetracyclic triterpenes	4.89
	β-cubebene	Sesquiterpenes	4.59
	n-hexadecanoic acid	Fatty acid	8.89
	9H-fluorene, 9-diazo-	Alkene	17.72
	Linolenic acid	Fatty acid	21.30
Ethanol	Stigmasterol	Tetracyclic triterpenes	4.01
	Linolenic acid, 2-hydroxy-1-(hydroxymethyl) ethyl ester (Z,Z,Z)-	Fatty acids	5.29
	Phytol	Terpene	8.31
	n-hexadecanoic acid	Fatty acid	11.46
	9H-fluorene, 9-diazo-	Alkene	11.83
	Linolenic acid	Fatty acid	22.72

### Enzyme activity

**Figure 3:**
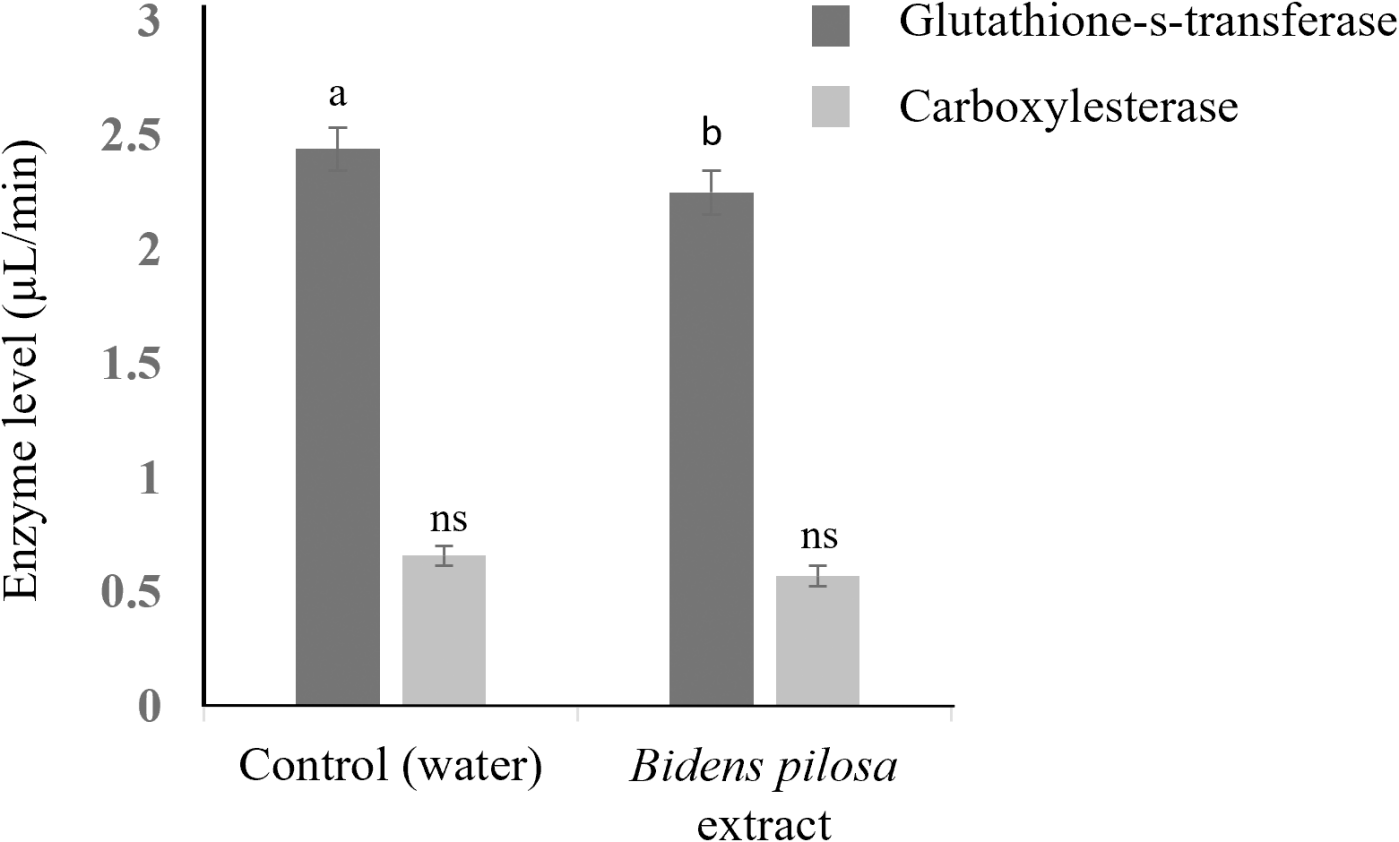
Enzyme levels of carboxylesterase and glutathione S-transferase in larvae of house flies treated with *Bidens pilosa* extract at a concentration of LC_90_ in ethyl acetate solvent. Different small letter indicates significantly different between treatment (*P* < 0.01), ns indicate not significantly different according to Paired t-test (*P* ≥ 0.05).

Larvae treated with a *B. pilosa* extract had lower CarE and GSTs activity. When compared to control, larvae treated with *B. pilosa* extract showed a significant decrease in GSTs activity (*t* =10.97, *df* = 4, 15, *P* = 0.0004) but not in CarE (*t* =1.61, *df* = 4, 15, *P* = 0.1838) (Fig. 3).

### Toxicity test on house flies in the poultry farm

The control of house fly in the poultry farm was assessed using the LC_90_ value of *B. pilosa* extracts in ethyl acetate solvent obtained from the feeding activity experiment. The findings showed that *B. pilosa* extract was effective in preventing house fly eggs from being laid, since eggs were not detected in the manure after spraying *B. pilosa* extract for 1–3 days before sampling. Furthermore, *B. pilosa* extracts showed no pupal stage after one day of spraying (Table 6).

The total number of house fly larvae and pupae was observed to have decreased after three days of spraying, as compared to the control. On the third day following application, percentage reduction was 75.71%. Following a five-day period, it was observed that the number of eggs, larvae, and pupae in *B. pilosa* extract treatment was lower than that of control (44.99% reduction). House fly eggs were not found by day 7. Percentage reduction of immature stages decreased to 10.54% (Table 6).

## Discussion

### Influence of extraction solvents on toxicity

The results demonstrated that the effects of *B. pilosa* extract differed depending on the solvent used during the extraction procedure and the precise immature stage of the house fly. For example, the hexane and ethyl acetate extracts of *B. pilosa* had high ovicidal activity, while they had low larvicidal activity. Contrastingly, strong larvicidal activity occurred when using high-polarity solvents (ethanol solvents).

These results align with the findings of Ardburai, Thongphak & Tangkawanit (2024), who reported that the larvicidal efficacy of plant extracts against the house fly is highly dependent on the extraction solvent. In our study, the ethyl acetate extract of *B. pilosa* exhibited potent larvicidal activity, with an LC_5__0_ value lower than that of several other plant species tested by Ardburai, Thongphak & Tangkawanit (2024), including *Alstonia scholaris*, *Murraya paniculata*, and *Citrus aurantium*. However, its efficacy was less than that of commercial neem and tobacco extracts (Kidtakhu et al., 2023).

### Chemical profile and stage-specific insecticidal efficacy

Consistent with the findings of Tiwari (2015), our GC-MS analysis confirmed that the chemical profile of an extract is highly dependent on the solvent used. Consequently, the three most potent extracts hexane, ethyl acetate, and ethanol were analyzed, revealing a diverse array of compounds in each (Table 5). Four main bioactive chemicals linolenic acid (fatty acid), 9H-fluorene, 9-diazo- (alkene), phytol (terpenoid) and stigmasterol (triterpenes), were present in different amounts across all solvents. These findings of bioactive compounds are different to previous reports in terms of quantity and type of compound. Khanal, Rana & Raut (2019) reported that an ethanol extract of the aerial parts of *B. pilosa* contained compounds from the tannin, alkaloid, saponin, and terpenoid groups. In contrast, Idris et al. (2023) identified fatty acids, phenolic acids, coumarins, furanocoumarins, and flavonoids as the primary constituents of *B. pilosa* ethanol extracts. Additionally, Ajanaku et al. (2018) observed that hexane extracted alkaloids and flavonoids, whereas ethyl acetate was effective in extracting steroids, tannins, and phenols. Xuan & Khanh (2016) reported that there were approximately 301 compounds in *B. pilosa*. Significant variation in the concentration of bioactive chemicals can be caused by a variety of factors, such as plant organs, phenological stage, genetic profile, and environmental abiotic or biotic factors, such as growing site, light, temperature, radiation, soil salinity and dryness, infections, and herbivore attacks (Çirak & Radusiene, 2019).

**Ovicidal activity,** the efficacy of *B. pilosa* extract differs according to the various functions performed by bioactive components at each phase of the house fly’s immaturation. Our results revealed a high ovicidal activity in the hexane and ethyl acetate extracts and GC-MS analysis revealed four major chemicals: β-cubebene, 9H-fluorene, 9-diazo-, and linolenic acid. Currently, there have been no reports of 9H-fluorene, 9-diazo- and linolenic acids affecting insect eggs, whereas the other two main compounds have been reported as having ovicidal activity. β-cubebene has been shown to interfere with the hatching and development of eggs and the particular mechanisms involve disruption of the control of hormones (Dávila et al., 2023). The ovicidal effects of β-cubebene from essential oils derived from the leaves of *Piper aduncum* L*.*, *P. gaudichaudianum* Kunth, *P. malacophyllum* Trin., *P. marginatum* Jacq., and *P. tuberculatum* Jacq. against *Tibraca limbativentris* (TIBRLI) were reported by Krinski & Foerster (2016). Moreover, the β-Cubebene molecule from *P. nigrum* has ovicidal effect on tick eggs (*Rhipicephalus microplus* (Canestrini, 1888)) rendering them unable of develop into larvae (Vinturelle et al., 2017). Phytol from various plants has been reported to influence egg hatchability. Adesina (2022) reported that high phytol (22.57%) from the ethyl acetate extract of *Datura metel* L. affected the egg hatchability of the cowpea weevil (*Callosobruchus maculatus* (Fabr.)). Apart from insects, a significant ovicidal effect on the decrease in hatching with 56.04% of mites (*Tetranychus urticae* (Koch)) was observed using phytol from *Tagetes patula* L. ethanolic leaf extract (62.72% phytol) (Ismail, Tag & Rizk, 2019). β-cubebene and phytol belong to the terpene group. According to Mazzini & Baiocchi (1983), the ovicidal effect of terpenes results from their capacity to penetrate the eggs *via* aeropyles present on the eggshell surface. Based on this finding, exposure to the extracts during the egg stage may result in egg abnormalities during embryogenesis and may lead to incomplete development.

**Table 6:** House fly population and percentage reduction following the application of *Bidens pilosa* extract in a poultry farm.

**Day**	**Egg**		**Larva**		**Pupa**		**Immature stage**		**% reduction** ^2/^
	**Control**	**Treatment**	**Control**	**Treatment**	**Control**	**Treatment**	**Control**	**Treatment**	
0	0 ± 0^c1/^	0 ± 0^b^	219.5 ± 18.94^a^	205.75 ± 27.03^a^	0 ± 0^d^	0 ± 0^c^	219.5 ± 18.94^b^	205.75 ± 27.03^a^	–
1	16.25 ± 15.54^bc^	0 ± 0^b^	209.5 ± 30.29^a^	153.25 ± 8.26^b^	1 ± 2^d^	0 ± 0^c^	229 ± 39.89^b^	153.25 ± 8.26^b^	28.61
3	34.25 ± 26.51^b^	0 ± 0^b^	164.5 ± 33.55^b^	34.25 ± 6.99^d^	72.25 ± 8.73^b^	25.75 ± 9.53^b^	263.5 ± 27.23^a^	60 ± 6.97^d^	75.71
5	81.75 ± 7.18^a^	27.25 ± 12.5^a^	84.5 ± 12.81^c^	45.75 ± 13.57^d^	88.75 ± 5.73^a^	58.5 ± 13.30^a^	255 ± 18.34^a^	131.5 ± 24.18^bc^	44.99
7	0 ± 0^c^	0 ± 0^b^	100 ± 12.11^c^	89.25 ± 17.17^c^	27 ± 4.54^c^	17.25 ± 6.23^a^	127 ± 13.08^c^	106.5 ± 20.24^c^	10.54
F-value	23.20	19.01	28.04	80.02	249.65	37.85	25.21	31.81	
df (treatment, error)	4, 15	4, 15	4, 15	4, 15	4, 15	4, 15	4, 15	4, 15	
*P*-value	0.0000	0.0000	0.0000	0.0000	0.0000	0.0000	0.0000	0.0000	
CV	53.36	102.57	28.04	15.42	13.69	38.59	10.10	14.61	

**Notes.**

^1/^ Within each column, Mean ± SD followed by the same small letter indicate not significantly different according to Fisher’s Least Significant Difference (LSD) (*P* ≥ 0.05).

^2/^ Percentage reduction calculated using Henderson & Tilton’s formula (1955).

**Larvicidal activity,** hexane and ethyl acetate extracts of *B. pilosa* showed strong larvicidal efficacy through feeding methods, whereas ethanol extract had high potential in the contact method. The results of GC-MS analysis indicate that linolenic acid, 9H-fluorene, 9-diazo-, β-cubebene, and n-hexadecanoic acid were the main components of *B. pilosa*.

According to Krzymańska (1976), linolenic acids have an impact on insect feeding habits, mortality, and fertility. Additionally, they impact insect larval physiology and fatty acid metabolism. Previous reports indicated that linolenic acid has high larvicidal activity in many insects, such as *Culex quinquefasciatus* Say, 1823 (Melo et al., 2018), *Aedes aegyptii (Diptera)*, *Helicoverpa zea (Boddie)* (Noctuidae), *Lymantria dispar* (Lepidoptera), *Malacosoma disstria* Hübner, 1820 (Lepidoptera), *Orgyia leucostigma* (J. E. Smith) (Lepidoptera) (Ramsewak et al., 2001), *C. maculates* (Coleoptera) (Adebowale & Adedire, 2006), and *Liposcelis bostrychophila* (Psocodea) (Green, 2011). Additionally, linolenic acid has an effect on the growth and development of insect larvae of *S. frugiperda* (Ramos-López et al., 2012) and *Creatonotus gangis* (Linnaeus, 1763) (Mondal & Chakraborty, 2017). Melo et al. (2018) reported that linolenic acid affects metabolism and the morphology of the midgut and fat body and causes a significant increase in acetylcholinesterase activity of *C. quinquefasciatus* larvae.

Larvae are toxically affected by β-cubebene. According to Chung et al. (2009), β-cubebene in *Dendropanax morbifera* oil (4.19%) has demonstrated a significant toxic impact against *A. aegypti* L. larvae (early fourth stage). Additionally, β-cubebene from *Salvia splendens* oil (22.9%) has shown a high inhibitory larvicidal activity against *A. albopictus* larvae (Mathew & Thoppil, 2011). β-cubebene is a terpene; according to Amos et al. (1974), terpenes have an impact on insects’ development and function similarly to hormones that regulate growth. Terpenes also had an effect on the antifeedant activity of *Tribolium castaneum* (Herbst, 1797) (Kim et al., 2010), *Sitophilus zeamais* (Motschulsky) (Yildirim, Emsen & Kordali, 2013), and *Leptinotarsa decemlineata* (Say) (Rodilla et al., 2008). Terpenes exhibit larvicidal effects by inhibiting the sterol carrier protein (AeSCP-2) in the larval midgut, as shown by Kumar et al. (2012). This disruption interferes with the physiological processes of cholesterol absorption and transport in insects, ultimately preventing their growth and reproduction (Li, Lan & Liu, 2009).

Compared to dipping approaches, feeding had a stronger larvicidal effect on the larval stage (Table 2). The feeding method showed significantly higher toxicity (72–100% mortality) compared to the dipping method (12–72% mortality) for all solvent (Table 2). This indicates that the *B. pilosa* extract is more effective when ingested, which superior strategy for larval control. These results suggest that while contact toxicity exists, oral administration maximizes the extract’s insecticidal potential, making it highly suitable for practical application in breeding substrates. This result suggested that *B. pilosa*’s bioactive components are more harmful to the digestive system than to other systems. The feeding method delivers toxins directly to the highly permeable midgut for efficient systemic absorption, while the dipping method is hindered by the insect’s waxy, less permeable cuticular barrier. However, in the experiment using the feeding method, the house fly larvae were transferred into the larval container and given time to survive after the *B. pilosa* extract was mixed with the larval food in the feeding container. This means that the experimental larvae also showed the contact effects of plant extract in addition to the primary effect of ingestion. Nevertheless, the concentration of plant extract after being combined with the larval meal decreased when compared with the dipping experiment.

The volatile chemicals 9H-fluorene, 9-diazo- were also detected in *B. pilosa*. These substances have larvicidal activity (100%) on the third larval stage of *C. quinquefasciatus* (Singh et al., 2017). However, their mode of action on insects is unknown.

**Pupicidal activity,** the results revealed that *B. pilosa* hexane extract had strong pupicidal activity. It’s primary bioactive components, as determined by GC-MS analysis, were 9H-fluorene, 9-diazo-, followed by linolenic acid, β-cubebene, and n-hexadecanoic acid.

Linolenic acid has been reported to have an effect on adult emergence (Mondal & Chakraborty, 2017). Vanderzant, Kerur & Reiser (1957) reported that linolenic acid had an effect on the adult emergence of *Pectinophora*. Additionally, Ramos-López et al. (2012) found that linolenic acid from *Ricinus communis* hexane leaf extract reduced the pupal weight of *S. frugiperda*.

β-cubebene is a sesquiterpene (Kerebba et al., 2019). Guillet et al. (2000) reported that sesquiterpenes affect insects by reducing glutathione levels, leading to significant mortality. According to Cárdenas-Ortega et al. (2015), β-cubebene derived from *S. ballotiflora* oil exhibits insecticidal properties against *S. frugiperda*. Additionally, β-cubebene extracted from *Perilla frutescens* (L.) Britton leaves have shown mortality effects on *Dermestes maculatus* DeGeer pupae (Ye et al., 2015). Furthermore, *Lippia origanoides* Kunth oil, which contains high levels of β-cubebene, demonstrated insecticidal activity on the pupal stage of *S. frugiperda* at a low concentration of 0.5%, causing failure to reach the pupal stage, preventing adult emergence, and resulting in mortality (Sombra et al., 2020).

n-Hexadecanoic acid acts as a free fatty acid, playing a vital role in the structure of cell and organelle membranes and serving as an essential energy source (Stanley-Samuelson et al., 1988; Soulages & Wells, 1994). Amala et al. (2021) reported that n-hexadecanoic acid and n-octadecanoic acid from *Epaltes divaricata* (L.) extracts exhibited significant growth inhibition, enzyme inhibition, larvicidal activity, and midgut toxicity against *A. aegypti* and *S. litura*. Additionally, studies have shown that n-hexadecanoic acid reduces the adult emergence of insects such as *S. frugiperda* (Sharanappa et al., 2024), *S. litura* and *H. armigera* (Jeyasankar & Chinnamani, 2017), and *Zeugodacus cucurbitae* (Coquillett) (Puri, Singh & Sohal, 2022).

### Enzyme activity

This study investigated the activities of two detoxification enzymes, CarE and GSTs. The *B. pilosa* extract selectively inhibited GSTs activity while having no significant effect on CarE activity. CarE, and GSTs contribute toward metabolic resistance to chemical pesticides (Hemingway et al., 2004). During phase I of the detoxification process, CarE modifies xenobiotics (foreign compounds) by oxidizing, hydrolyzing, and reducing them (Yu, 2008; Aioub & Ashour, 2023). This enzyme catalyzes the breakdown of insecticides into less toxic metabolites (Feng & Liu, 2020). It’s possible that *B. pilosa* extracts had no influence on the phase I detoxifying enzyme process because our results showed no effect on CarE when compared to the control.

GSTs are enzymes that facilitate the detoxification of xenobiotic substances in an organism in phase II of detoxification by catalyzing the conjugation of glutathione (GSH). GSTs protect against oxidative stress induced by exposure to xenobiotics; they help maintain redox homeostasis by scavenging reactive oxygen species (ROS) (Koirala, Moural & Zhu, 2022). This process helps to neutralize potentially harmful chemicals, allowing insects to survive.

The significant reduction in GSTs activity observed in this study provides a clear mechanistic basis for the insecticidal efficacy of our extract. GSTs are crucial for detoxifying xenobiotics, and by inhibiting their function, the bioactive compounds in the extract likely compromise the insect’s primary defense system. This inhibition would lead to an accumulation of toxic compounds within the insect, inducing severe oxidative stress. This uncontrolled oxidative stress is known to cause widespread damage to vital cellular components, providing a direct mechanistic link between the observed GSTs inhibition and the resulting mortality (Lushchak et al., 2018). Future studies could further confirm this pathway by measuring levels of glutathione and specific markers of oxidative damage

Pavlidi, Vontas & Van Leeuwen (2018) reported that GSTs contribute significantly to insecticide resistance through two main mechanisms, including metabolic detoxification and sequestration of insecticides. Insect species have developed resistance to commonly used insecticides through enhanced detoxification of enzyme activity. Increased activity of GSTs and esterase has been linked to resistance against organophosphates and pyrethroids in various pest populations (Nardini et al., 2012; Koffi et al., 2014; Aioub & Ashour, 2023). The decrease in GSTs found in this investigation suggested that house flies were not resistant to *B. pilosa* extracts.

The present study shows that extracts from *B. pilosa* reduced the activity of GSTs. According to Gökçe (2023), bioactive chemicals present in various plant extracts have the ability to inhibit GSTs enzymes. For instance, anthraquinone compounds found in some plants have been demonstrated to significantly decrease GSTs activity. These compounds bind to GSTs and form stable complexes, which limit their enzymatic function. Additionally, Ahmad & Srivastava (2008) reported that fatty acids inhibit GSTs activity by working as ligands that prevent enzyme function. The GSTs gene family encodes genes that involve detoxification enzyme GSTs (Nebert & Vasiliou, 2004). Rand et al. (2015) suggested that a smaller number of GSTs genes were correlated with increased sensitivity to pesticides of insects (as demonstrated in the honeybee), potentially leading to reduced vitality and increased pesticide susceptibility.

### Toxicity test to house flies in the poultry farm

The results showed that *B. pilosa* extract effectively decreased the number of house flies in the poultry farm within three days of application. No fresh house fly eggs were discovered in the treatment areas throughout this time. House fly oviposition inhibition may be one of the causes. According to Ajileye & Muse (2006), *Bidens pilosa* has an anti-ovipositional effect in *A. aegypti*. The possibility of repelling other insects, such *Armadillidium vulgare* (Latreille), was also mentioned (Ishida et al., 2023). Therefore, the extract from *B. pilosa* might prevent oviposition and deter adult houseflies.

After 3 days of the experiment, the number of houseflies slightly increased, indicating the degradation of the compound in the environment. This aligns with findings by Pavela (2014) and Essiedua, Adepoju & Ivantsova (2020), who reported that biological pesticides have a high biodegradation rate, making plant extracts more biodegradable and environmentally friendly compared to chemical insecticides. Tembo et al. (2018) noted that plant extracts can rapidly degrade due to environmental factors such as sunlight (photodegradation) and rainfall, which may wash away the extracts. Laleh et al. (2006) also reported that rising temperatures can accelerate the degradation of bioactive compounds. Although plant extracts are effective for controlling house fly populations, they have limitations, as bioactive compounds degrade easily. Thus, creating an effective formulation for this plant extract will be crucial to ensure its sustained effectiveness.

Limitation of this study primarily concerns concentration refinement rather than LC_5__0_ validity. The selected concentrations produced a statistically reliable Probit model with acceptable goodness-of-fit (Chi-square, *p* > 0.05), providing a dependable baseline for toxicological assessment. Although additional intermediate concentrations may further improve LC_5__0_/LC_9__0_ precision, the current design was sufficient to establish insecticidal effectiveness. Additionally, although the identified compounds, such as linolenic acid, have been reported as effective against house flies (Adebowale & Adedire, 2006; Xuan & Khanh, 2016), the potential effects on non-target beneficial insects were not examined in this study. Future research should therefore evaluate environmental safety, particularly on pollinators and natural enemies, to further support the development of this extract as a sustainable biopesticide.

## Conclusions

The efficacy of *B. pilosa* extract on house flies varies depending on the solvent used for extraction. Extracts using hexane and ethyl acetate induced significant mortality rates at the egg stage of house flies. The larvicidal bioassay results indicated that acetone and ethanol extracts exhibited strong larvicidal activity when applied *via* the dipping method, while hexane and ethyl acetate extracts demonstrated 100% larvicidal activity through the feeding method. Hexane, ethyl acetate, and acetone extracts of *B. pilosa* caused significant mortality (>90%) in the pupal stage of house flies. Although other treatments did not show significant effects on insect mortality, they influenced the developmental periods of house flies in subsequent stages. This study also found that *B. pilosa* extracts impacted the sex ratio of adult flies, resulting in a higher proportion of males than females. Additionally, treatment with *B. pilosa* extracts reduced GSTs activity in larvae, although no effect was observed on CarE activity. The most effective treatments for each stage of the house fly were analyzed using GC-MS to identify bioactive compounds, revealing β-cubebene, 9H-fluorene, 9-diazo-, linolenic acid, and stigmasterol as the primary active compounds. The lethal concentration (LC_50_ and LC_90_) for the most effective *B. pilosa* extract against each immature stage of house flies was estimated. The LC_90_ results indicated that ethyl acetate extracts, particularly when applied through the feeding method, were highly effective in controlling house flies within 1-3 days in poultry farms. Additionally, *B. pilosa* extracts may have potential applications for controlling house flies in urban and veterinary settings during their immature stages.

While this plant extract shows significant potential as a biopesticide, its viability is currently limited by short-term effectiveness, potential environmental degradation, and practical application challenges. Therefore, future research should focus on assessing the toxicity of this extract against key non-target organisms to determine their environmental safety and viability as a sustainable biopesticide. Overcoming these hurdles is essential to validate its use in an integrated pest management program.

##  Supplemental Information

10.7717/peerj.21544/supp-1 Supplemental Information 1 Raw data

10.7717/peerj.21544/supp-2 Supplemental Information 2 AI Prompt AI Prompt for Gemini AI (Google)

## Additional Information and Declarations

### Data Availability

The following information was supplied regarding data availability: The raw measurements are available in the Supplemental File.
