# Supplementary material for: Control efficacy of Bidens pilosa L. extract against immature stages of Musca domestica L. (Diptera: Muscidae): laboratory and semi-field evaluations
Source: PeerJ. 2026 Jul 17;14:e21544. doi: 10.7717/peerj.21544 (PMC13383962; doi:10.7717/peerj.21544)
Supplement: Supplemental Information 2 — AI Prompt for Gemini AI (Google) [file peerj-14-21544-s002.docx]

AI **Prompt** Gemini AI (Google):

**Prompt:** Please create the schematic illustrations using this information "The experiment was carried out in an open rearing house with dimensions of 6$\times$50$\times$2 m. The lower portion of the house was built with concrete, while the upper part was enclosed with a wire mesh extending to the ceiling. There were 2 long shelves in the poultry house with a total of 312 chickens (4 strains). Each chicken was raised in a small cage on the shelf in the rearing house. On the left side, Super Haoka chickens were in the upper cages, and Barred Plymouth Rock chickens were in the lower cages. On the right side, White Leghorn stayed in the cage above, and Rhode Island Red chickens were in the lower cages. Food and water were provided in the cage every day. On the floor beneath, chicken excrement was the breeding source for the house fly breeding colony."
